# A rare case of ovarian hemangioma in a 30-year-old nulligravid woman

**DOI:** 10.1016/j.radcr.2022.10.100

**Published:** 2022-11-30

**Authors:** Harry Ramos, Nithin Thummala, Suzanne M. Jacques, Radhika Gogoi

**Affiliations:** a Wayne State University School of Medicine, 540 E. Canfield Ave, Detroit, MI 48201, USA; b Karmanos Cancer Institute, 4100 John R St, Detroit, MI 48201 USA

**Keywords:** Ovarian hemangioma, Ovary, Hemangioma, Cavernous-type, Capillary-type, Vascular tumor

## Abstract

A 30-year-old nulligravid woman with a history of polycystic ovarian syndrome presented for evaluation of left adnexal mass. The patient was referred to the gynecologic oncology clinic after endorsing signs of abdominal pain for a month and the pelvis ultrasound demonstrated hypoechoic solid mass in the left ovary. Magnetic resonance imaging with T1- and T2-weighted images demonstrated progressive centripetal “filling-in” of the mass suggesting a unique variation of malignant ovarian mass, similar to what is seen in hepatic hemangioma. Upon resection of the ovarian mass, pathology reported that the mass was filled with numerous small blood vessels with single later of endothelial cells confirming the diagnosis of ovarian hemangioma, capillary-type—a rare finding.

## Introduction

Ovarian hemangiomas (OH) are a rare gynecologic finding, with less than 60 reported cases in the literature [1], [2], [3], [4], [5]. OH are generally incidental findings during surgery but can also present as symptomatic adnexal torsion, or ovarian lesions mimicking findings suggestive of ovarian neoplasms [3]. In these cases, clinical findings such as ascites, Meigs syndrome, and elevated CA-125 have been reported as usual manifestation of OH. From an imaging standpoint, OH can be misinterpreted as benign calcifications of the ovary [2].

In this case report, we present a 30-year-old female with a history of polycystic ovary syndrome (PCOS) who underwent a diagnostic laparoscopy due to inconclusive left adnexal mass findings on ultrasound (US) and magnetic resonance imaging (MRI). Radiology review suggested a unique presentation of the left adnexal mass with MRI findings similar to that of a hepatic hemangioma. Specifically, a progressive centripetal enhancement seen on post-contrast imaging in a typical liver hemangioma which is also seen in the MRI images of this patient's left ovary. Thus, we report an incidental finding of an OH which mimics a presentation of a hepatic hemangioma highlighting the unique radiologic characteristics of this pathology in the ovary

## Case presentation

A 30-year-old nulligravid woman with a history of PCOS presented to the clinic for evaluation of adnexal mass. The patient was referred by her primary care physician due to increasing left sided abdominal pain for the past month. During the visit, the patient complained of mild pain in that location but denied any fever, chills, chest pain, and shortness of breath. Of note, the patient does not have a family history of breast, cervical, and colon cancer. Despite her PCOS, the patient reported regular menstrual cycle and an unremarkable gynecologic history. Otherwise, the patient had unremarkable physical exam limited slightly by her BMI (BMI = 40 kg/m^2^). TSH and HgbA1c were within normal limit. Tumor markers were CA-125 is 12 units/mL and HE4 is 31 pmol/l.

**Fig. 1:**
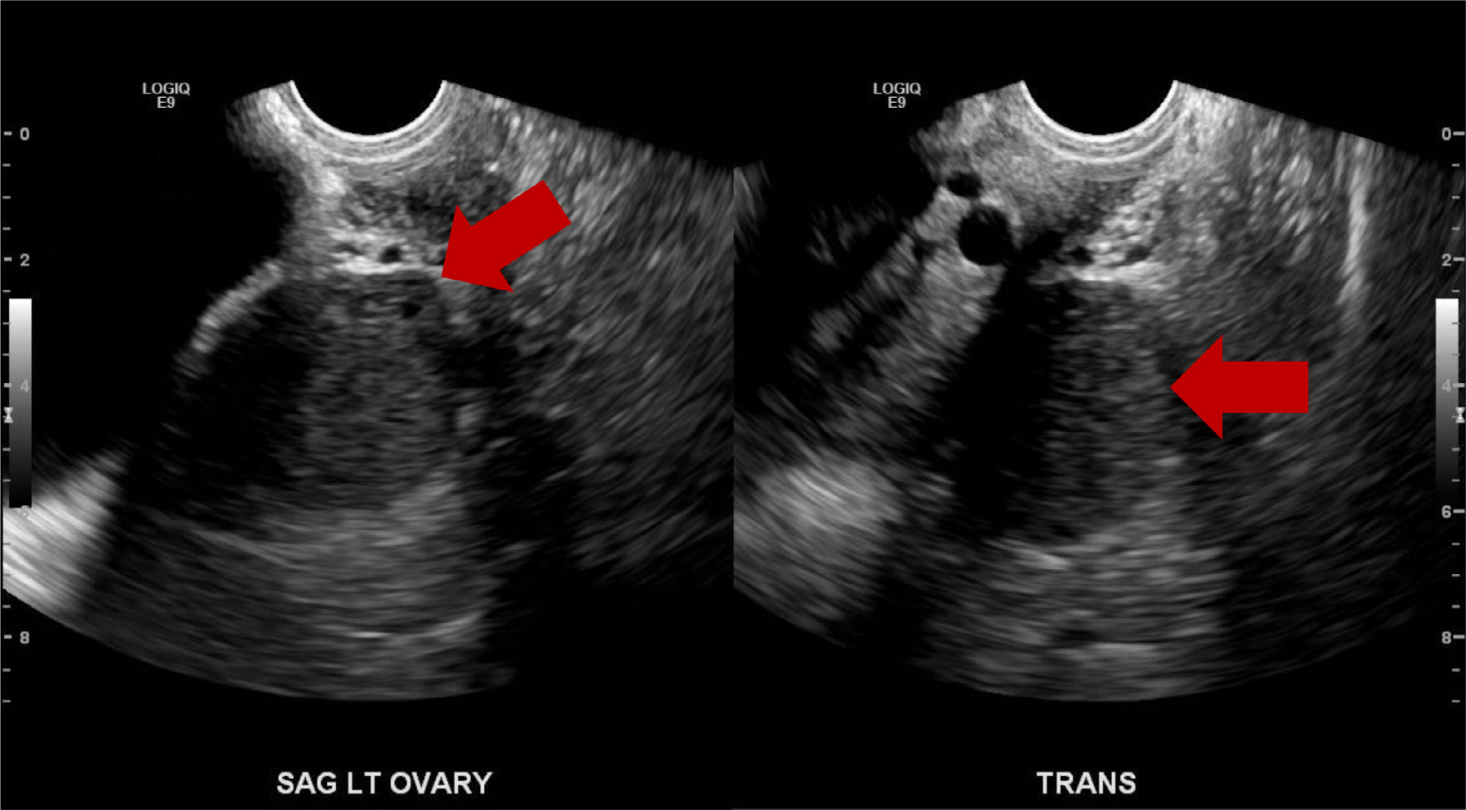
(A and B) Transvaginal ultrasound of left ovaria (in grayscale). Both sagittal and transverse images demonstrate a hypoechoic, solid mass in the left ovary.

**Fig. 2:**
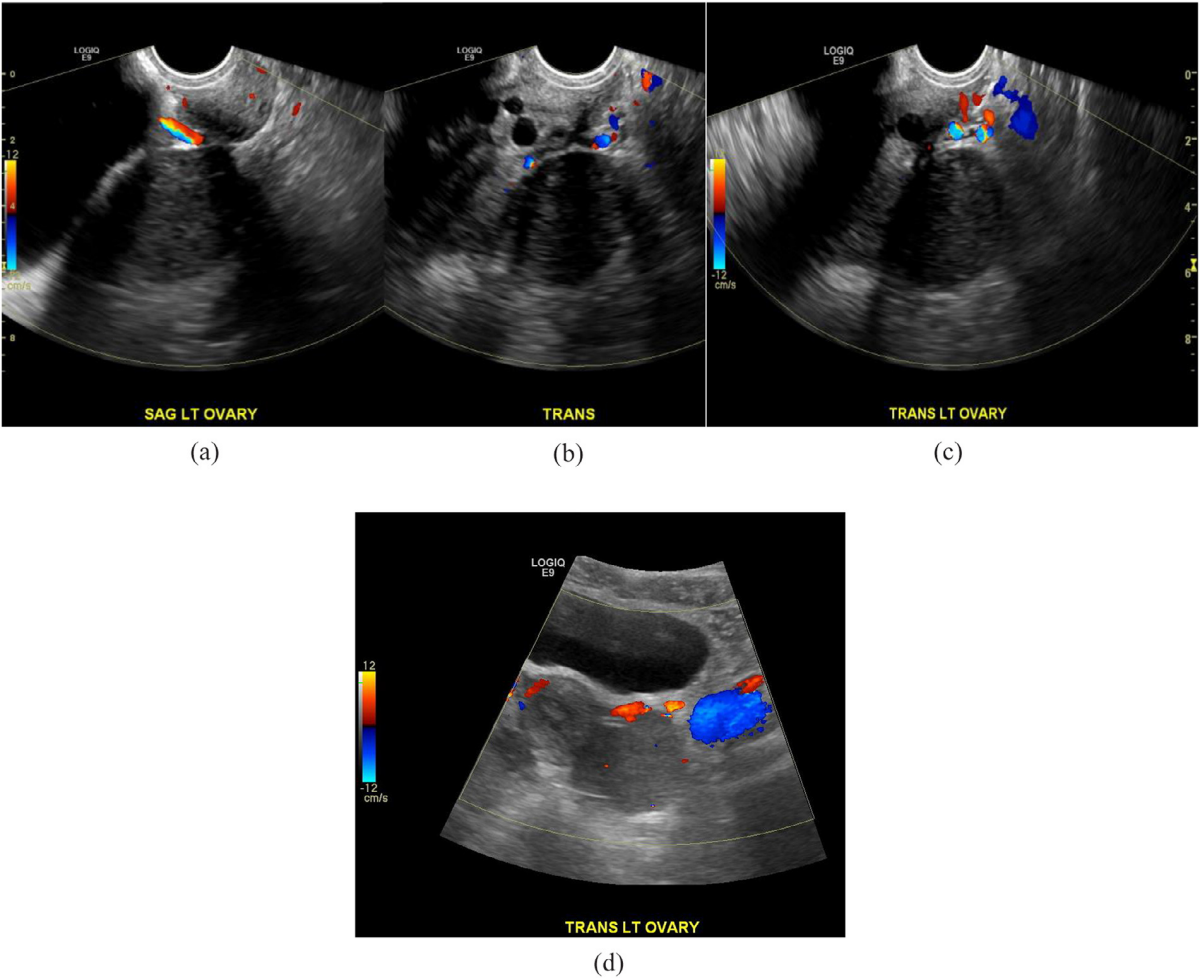
(A-C) Transvaginal ultrasound of left ovaria showing no significant flow. (D) Transabdominal ultrasound of the left ovary showing very minimal flow.

**Fig. 3:**
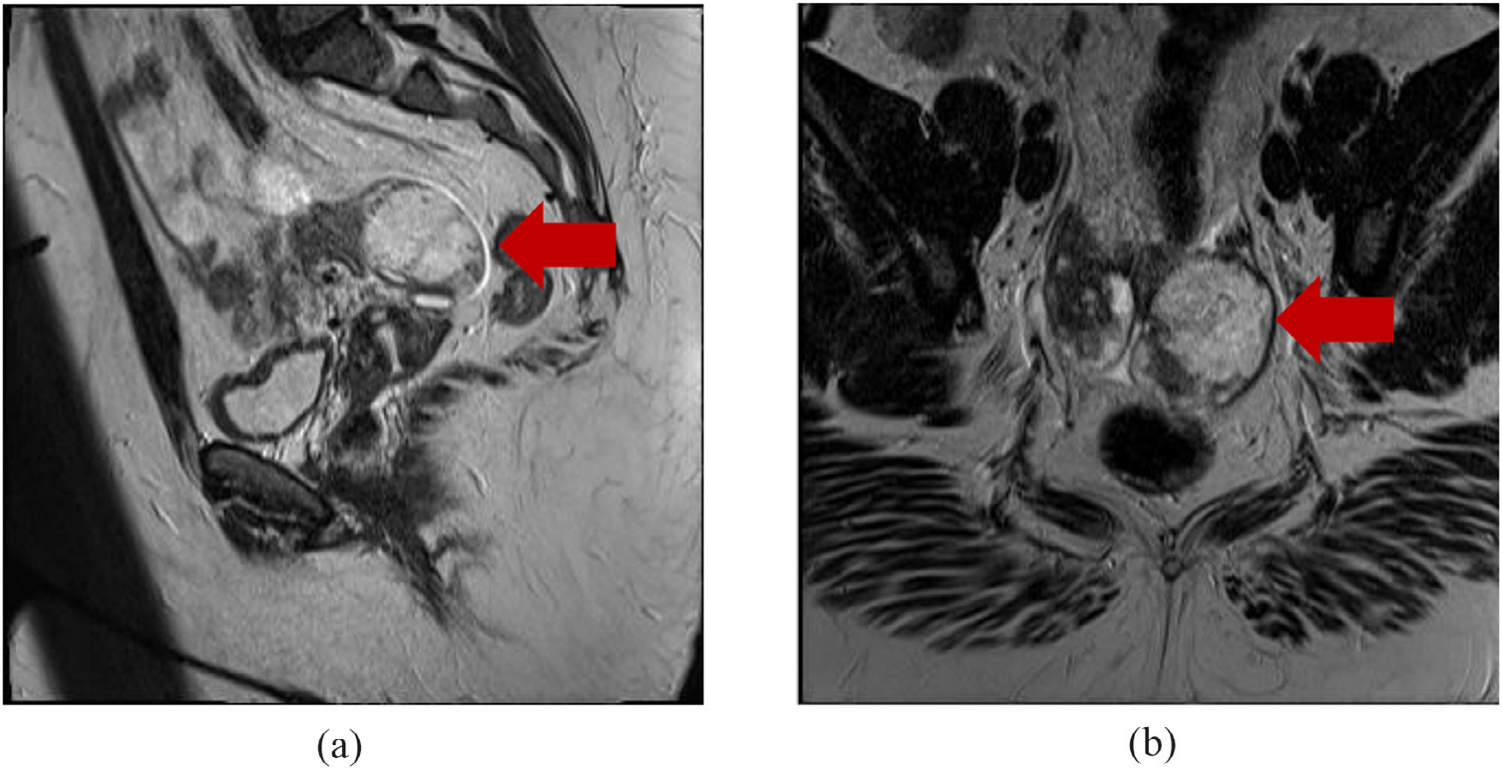
(A) MRI of the pelvis. Sagittal T2-weighted image showing a hyperintense left ovarian mass. (B) MRI of the pelvis. Axial T2-weighted image showing a hyperintense left ovarian mass.

**Fig. 4:**
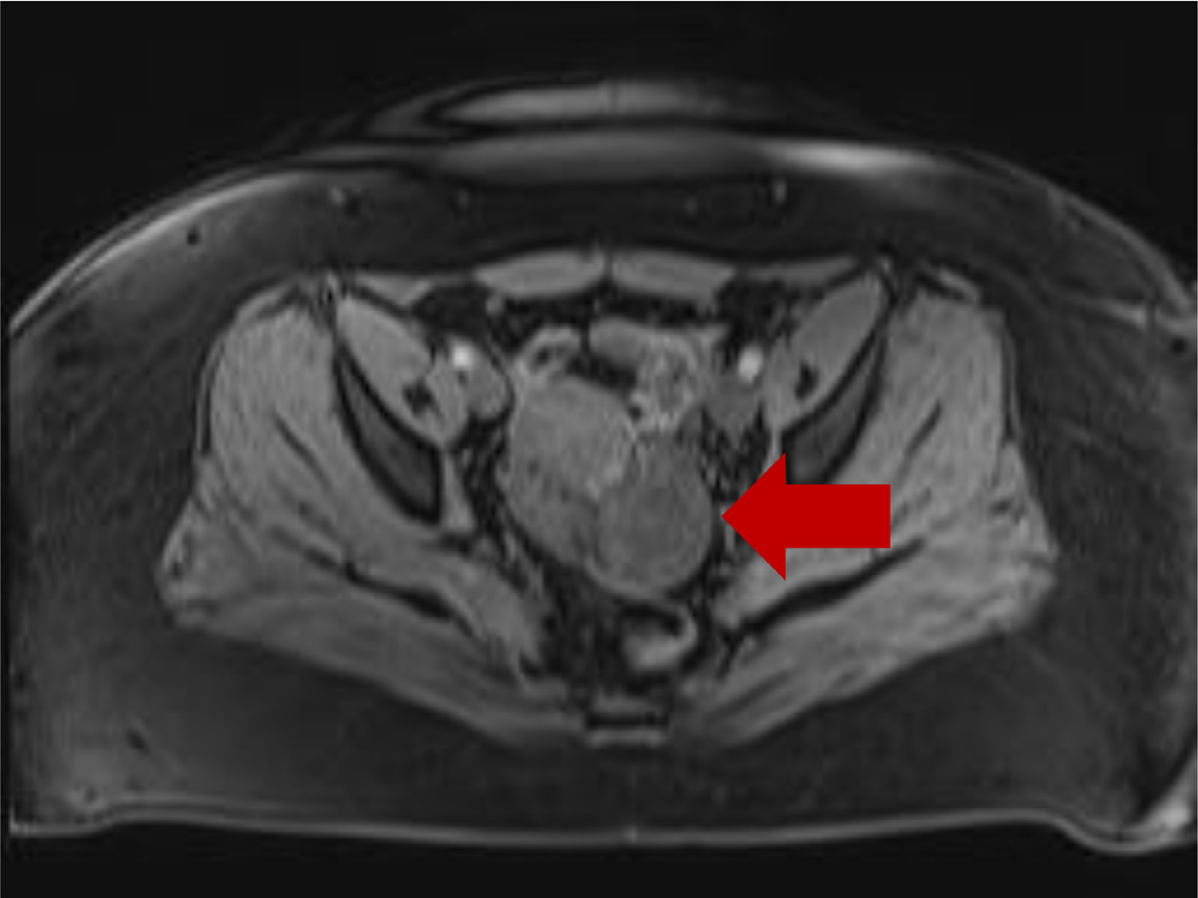
MRI of the pelvis. Axial T1-weigthed image demonstrates a minimally hypointense left ovarian mass.

**Fig. 5:**
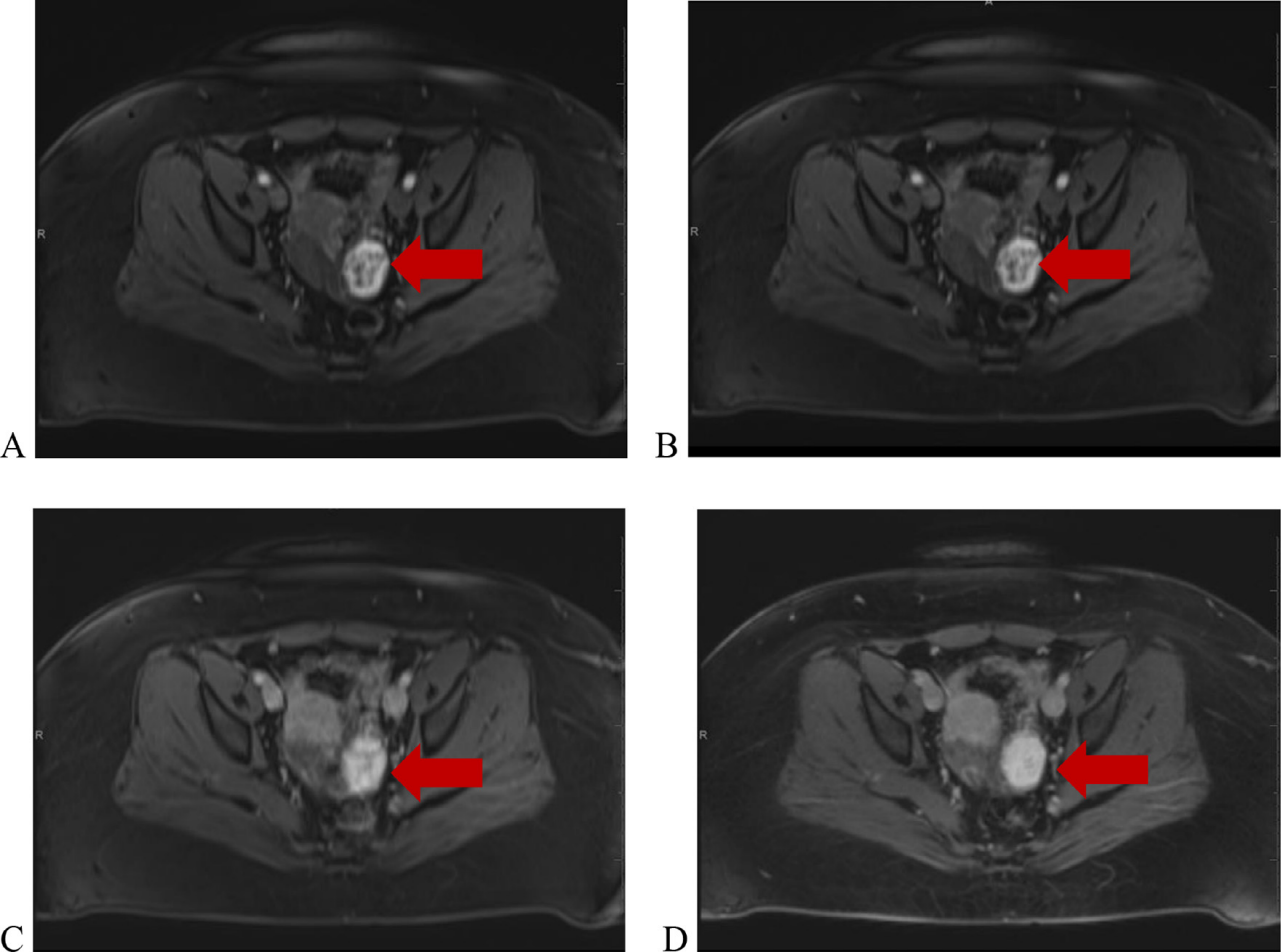
(A-D) MRI of the pelvis. Axial post-contrast T1-weighted images demonstrate avid enhancement with progressive centripetal “filling-in” at 30, 60, 90, and 120 seconds.

After presenting to the clinic, US and MRI of the pelvis were performed. US findings indicated enlargement of the left ovary without evidence of left ovarian follicles with radiological concern of adnexal mass. Most notably, US demonstrated a hypoechoic solid mass in the left ovary (Figs. 1A & B). A Doppler study was also performed and it showed no significant flow (Figs. 2A-D). MRI was recommended and performed for further diagnostic work up. T2-weighted images depicted a hyperintense left ovarian mass and (Figs. 3A & B) T1-weighted images also demonstrated a minimal hypointense signal (Fig. 4). Post-contrast T1-weighted images obtained at 30, 60, 90, and 120 seconds demonstrated avid enhancement with progressive centripetal “filling-in” of the mass suggesting a unique variation of malignant ovarian mass (Figs. 5A-D). With these findings, patient was scheduled for a diagnostic laparoscopy.

A laparoscopic left salpingo-oophorectomy was performed. Upon entrance into the abdomen, a 4-5 cm in diameter cyst was noted in the left ovary. Mass was excised intact and sent to pathology. Pelvic washing was obtained for cytology. Otherwise, normal appearing uterus, bilateral fallopian tubes, and right ovary were noted during surgery. Due to a likely negative pressure pulmonary edema with frothy secretions, the patient remained intubated and was discharged 2 days after the operation. Post-op clinic visit was scheduled 6 weeks after and patient was recovering well.

## Discussion

Hemangiomas are benign vascular tumors that are usually asymptomatic and resolve spontaneously. They are most commonly found in the skin and the liver and are diagnosed from infancy to elderly. Further, hemangiomas are classified as capillary type (small blood vessels) and cavernous type (large blood vessels) [7]. Liver hemangiomas are most commonly of the cavernous type with an incidence rate of up to 20% [8]. In radiological images, liver hemangiomas have a pathognomonic appearance. MRI findings described these tumors as homogenous, well-demarcated lesions with very high signal intensity in T2-weighted images. In a CT scan, liver hemangiomas are typically hypodense on unenhanced images. When contrast medium is administered, these hemangiomas produce a pattern of enhancement with early peripheral nodular enhancement followed by centripetal filling in of the lesion on delayed phases [9]. From a pathology standpoint, the microanatomy of a well vascularized hemangioma correlates with the radiological description. With our patient, these similar—almost identical—radiological findings were described in the ovaries, where vascular tumors are not commonly found.

The ovary is a well vascularized organ of the female reproductive system. However, vascular tumors of the ovary—ovarian hemangiomas (OH)—are very rare with less than 60 cases reported in the literature. In fact, most cases of OH are incidental findings during surgery or autopsy [1]. OH does not have predilection for an age group. It has been found from patients ages 8-81-years-old [1], [2], [3], [4], [5], [6]. Diagnosing OH can be difficult as it can be asymptomatic or with findings that mimic that of an ovarian cancer. In addition, the difficulty of diagnosing ovarian vascular tumors in reproductive age women can be quite challenging. The cyclical nature of a female reproductive organs, as well as the asymptomatic and small size follicular changes that are associated with menstrual cycle, pose a challenge in properly identifying the existence of a growing vascular tumor in the ovary [10].

The etiology of OH remains unknown. However, OH can or has been described as a complication of stromal luteinization, stromal hyperplasia, and pseudo-Meigs’ syndrome [1,[11], [12], [13]. There are several hypotheses that explain the genesis of OH. One proposed hypothesis suggests that hormonal effects, such as in pregnancy, leads to growth and proliferation of OH [14]. Another hypothesis suggests that the presence of OH induces stromal luteinization by mass effect. This leads to increase production of androgen and unopposed conversion to estrogen, which trigger proliferation of the existing hemangioma [15]. Although the prevailing thought that hyperestrogenism is the growth stimulatory factor of vessels in OH, etiology remains controversial [16]. Interestingly, our patient's OH immunohistochemical staining for estrogen (ER) and progesterone (PR) is negative in its endothelial cells.

Given the vascular nature of an ovarian hemangioma, pelvic arteriovenous malformation (AVM) must also be considered in the differential diagnosis of patients with adnexal mass without other signs of malignancy. AVM are aberrant connections between arteries and veins and can be found anywhere in the body. AVM can be asymptomatic, or when multiple shunts are created within an AVM, its size enlarges, making the vessels weak and leak, which then acutely bleed. Depending on the AVM—its size and flow characteristics—treatment can be sclerotherapy or embolization, obviating the need for surgery [17].

Our patient had a clinical presentation of persistent localized left abdominal pain that triggered the pelvic US imaging of her primary care provider. This led to the findings of an adnexal mass. Additional MR imaging confirmed a left adnexal mass that was uniquely described similar to that of a liver hemangioma. Given the lack of family history of cancer and other symptoms such as ascites, weight loss, and high CA-125 levels, the clinical suspicion of ovarian cancer remained low, however, not ruled out. The American College of Obstetricians and Gynecologists recommendation is to surgically resect this tumor and send for pathology for definitive diagnosis and correlated treatment [18].

**Fig. 6:**
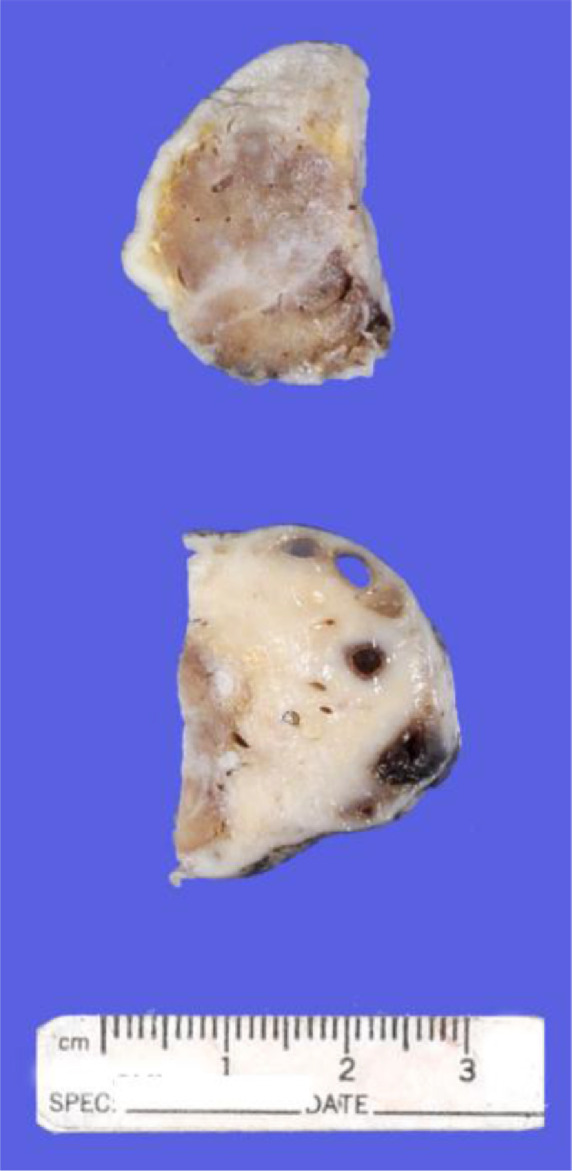
Gross anatomical photo of the patient's bisected left ovary (after formalin fixation and sectioning) demonstrating the hemangioma (above) and normal tissue (below).

The pathology of the adnexal mass correlates with the US and MR radiographic findings which allowed us to definitively diagnose the patient with an OH. Grossly, the specimen consisted of an ovary with attached fallopian tube weighing 24 g. The ovary measured 4.0 × 3.5 × 2.5 cm, and the outer surface was tan-gray and smooth to focally cerebriform. Sectioning revealed a 2 cm in diameter round well-circumscribed red-brown rubbery to spongy nodule comprising 50% of the ovary. The remaining ovarian tissue was unremarkable and included few small cystic follicles (Fig. 6). The fallopian tube was unremarkable.

**Fig. 7:**
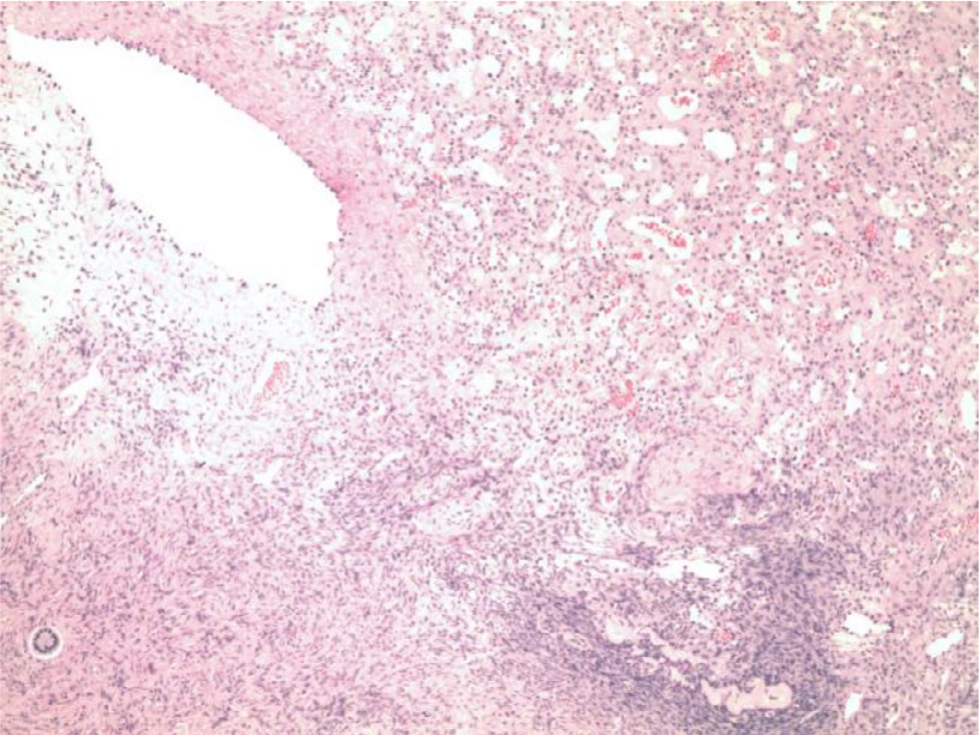
Microscopically, the ovarian nodule was composed of numerous small vascular spaces, consistent with ovarian hemangioma, capillary-type (H&E stain, 40×).

**Fig. 8:**
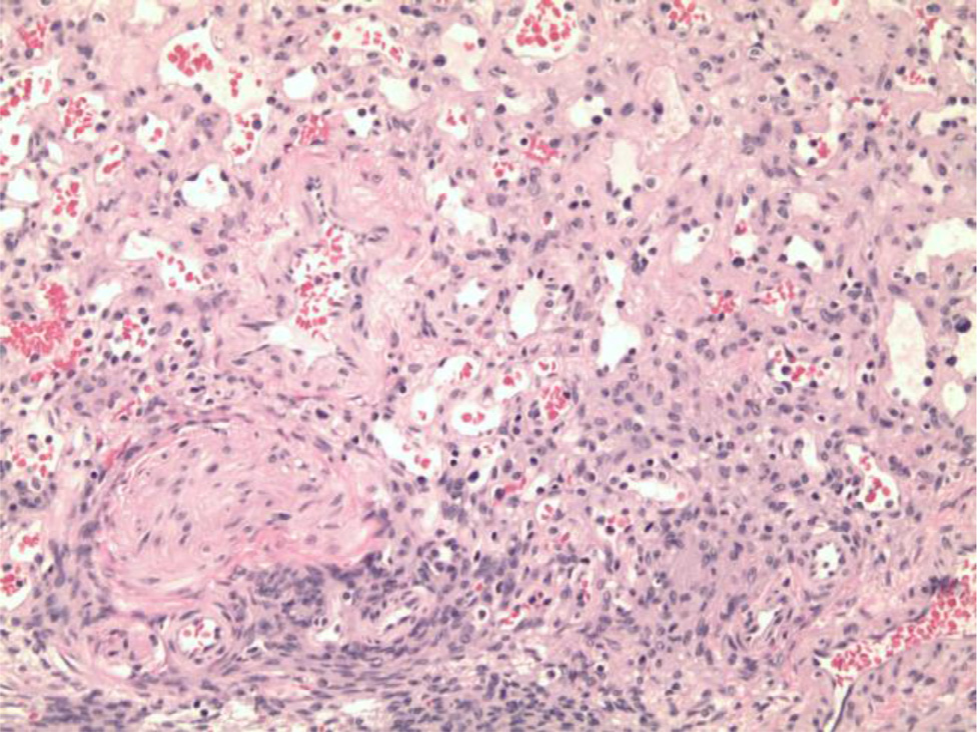
Higher magnification shows the vascular spaces to be lined by a single of endothelial cells and containing red blood cells (H&E stain, 100×).

**Fig. 9:**
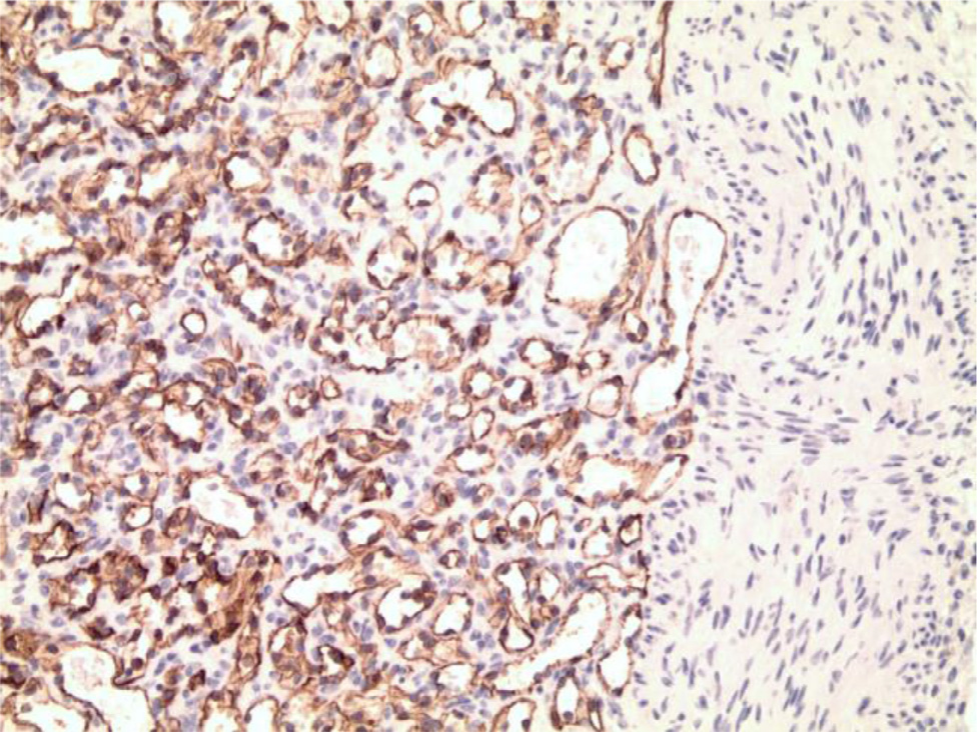
Immunohistochemical staining for CD31 is strongly positive in the endothelial cells (H&E, 100×).

Microscopic examination showed a vascular lesion composed of numerous small blood vessels lined by a single layer of endothelial cells and containing red blood cells (Figs. 7 and 8). There were few larger blood vessels with muscular walls. Only minimal stroma was present. No atypia, necrosis, or increased mitotic activity was seen. Immunohistochemical staining for CD31 was diffusely and strongly positive in the endothelial cells (Fig. 9), and immunohistochemical staining for pancytokeratin (AE1/AE3) was negative, confirming the vascular nature of the lesion. No inflammation, calcification, thrombi, or hemosiderin deposition was present. No additional teratomatous elements were identified. The uninvolved ovarian tissue contained small cystic follicles. No stromal luteinization was seen. The microscopic features and immunohistochemical staining are diagnostic of ovarian hemangioma, capillary-type.

OH vary in characteristics from anatomical and pathological presentations. Size ranging from 5 mm to 24 cm in width have been found. These vascular tumors are usually unilateral, but there have been some bilateral cases reported [16,19]. Similar to this patient, OH are usually found in the hilum of the ovaries, although it does not have predilection where it can form [19]. In addition, like any hemangiomas in other anatomical locations, there are 2 common types—cavernous or capillary [1]. Unlike the predominantly cavernous type found in most OH cases, our patient has the capillary-type, which is also the type of hemangioma found most commonly in the body [1].

From a radiological standpoint, the preoperative transvaginal US indicated solid mass in the left ovary. In the setting of PCOS, endometrioma was included in the differential diagnosis. However, there was convincing evidence with our MRI images that this adnexal mass carries a unique characteristic. Here, the T1 imaging revealed that the mass is being filled in with contrast which alludes to the vasculature nature of the tumor. Further, the T1-weighted MR images demonstrated a hypointense mass with markedly increased intensity on the T1-weighted images from 30 to 120 seconds. When compared to cavernous hemangioma found in the liver—a neoplasm that has a long transverse relaxation time compared with normal liver parenchyma—the patient's MR images were suggestive of radiologic diagnosis of an OH [20,21].

## Conclusion

Ovarian hemangioma is a rare diagnosis that is commonly discovered as an incidental finding during surgery or autopsy. Although there is variance in both age and clinical presentations, these benign tumors should be considered as a possible pathology of any adnexal mass. MR imaging of ovarian hemangioma is comparable to that of a hepatic hemangioma. This radiological similarity can be leveraged by clinicians in including this rare finding in their differential diagnosis and properly treat this vascular lesion.

## Patient consent

We obtained both verbal and written informed consent from the patient that her case will be used for publication.
